# The Psychogeriatric Patient in the Emergency Room: Focus on Management and Disposition

**DOI:** 10.1155/2014/413572

**Published:** 2014-03-10

**Authors:** Sherry Tang, Priyanka Patel, Jagdish Khubchandani, George T. Grossberg

**Affiliations:** ^1^School of Medicine, Saint Louis University, St. Louis, MO 63108, USA; ^2^DeKalb Community Service Board, Decatur, GA 30031, USA; ^3^Department of Physiology and Health Sciences, Ball State University, Muncie, IN 47306, USA; ^4^Department of Neurology & Psychiatry, School of Medicine, Saint Louis University, St. Louis, MO 63104, USA

## Abstract

*Background.* The growing geriatric population in the United States (US) has prompted better understanding of treatment of the elderly in the hospital and emergency room (ER) settings. This study examines factors influencing the disposition of psychogeriatric patients after their initial presentation in the ER. *Methods.* Data was collected on patients 65 years of age or older arriving at the ER of a large urban hospital in the USA (January 2009–December 2010). *Results.* Of the total subjects (*n* = 95) included in the study, majority were females (66.3%) with an average age of 75.5 years. The chief complaint for psychogeriatric patients coming to the ER was delirium (61.6%). Caucasians were significantly more likely than African-American patients to get a psychiatric consult (33% versus 9%). Patients with delirium were less likely than patients with other psychiatric complaints to get a psychiatric consult in the ER (1.2% versus 47.2%) and less likely to be referred to a psychiatric inpatient unit compared to patients with other psychiatric complaints (2.4% versus 16.7%). *Conclusion.* Even though delirium is the most common reason for ER visits among psychogeriatric patients, very few delirium patients got a psychiatric consultation in the ER. A well-equipped geriatric psychiatry unit can manage delirium and associated causes.

## 1. Introduction

According to the recent United States (US) census bureau report, the older population (65+ years of age) is projected to increase to 88.5 million by 2050, which is more than double the population of elderly people (40.2 million) in 2010 [1]. Due to the surge in the aging population, the US health care system will face increasing challenges in caring for older adults in the near future. Emergency rooms (ER) have been increasingly visited by older adults who present with complex medical conditions and who are more likely to have longer stays, requiring extensive testing, and treatment regimens [2].

The geriatric patients are a unique group and specific considerations should be included in the interventions for these patients. More specifically, psychogeriatric patients may have a variety of clinical presentations, including delirium, behavioral changes, mood disorders, and others. Impaired mental status occurs in approximately one quarter of all older patients presenting to the ER [3]. Delirium is a serious disturbance in a person's mental abilities that results in a decreased awareness of one's environment and confused thinking. The onset of delirium is usually sudden, often within hours or a few days. While several studies have been conducted that focus on the recognition and presentation of psychogeriatric patients in the ER [4–6], only a few studies discuss patient management and disposition after the initial ER visit, which are discussed below.

Lin and colleagues found that there were significant differences among elderly and nonelderly visitors to the ER based on demographic and clinical characteristics. Elderly visitors were more likely to have multiple psychotic disorders, mood disorders, dementia, or delirium and less likely to have schizophrenia and substance-related disorders. Transfer to a general hospital for the elderly in the psychiatric ER was associated with age and a greater number of previous psychiatric hospitalization [7]. In addition, older adults with alterations in mental status—particularly, alterations in consciousness and delirium, are at high risk for admission to an inpatient unit and institutionalization after discharge [8]. Finally, several studies of psychiatric patients regardless of their ages have reported factors such as danger to self, severity of psychosis, diagnosis of dementia, female gender, and referral to hospital by physician as favoring inpatient admission [6, 9, 10].

Inpatient admission of psychogeriatric patients to a psychiatric unit was specifically examined in a study showing that out of the 645 geriatric patients admitted to the inpatient psychiatry unit, 30% of the patients came from the emergency room and the rest from physicians in private practice or other departments or hospitals, with the majority from private practice [11]. The main reason for inpatient psychiatry admission was delirium, with endangering behavior to self or others as an important cause for admission [11].

Currently, there are no guidelines or recommendations concerning the indication for inpatient psychiatric admission [9–11]. Factors unique to each patient may lead to different treatment strategies. Understanding these factors may help ensure a more efficient and effective method of managing psychogeriatric patients in the ER setting. Therefore, the purpose of this study was to scientifically examine the presentation, management, and disposition of psychogeriatric patients 65 years of age or older who present to the ER. In addition, we also intended to assess factors associated with psychiatric consultation in ER for older patient population.

## 2. Methods

### 2.1. Study Design

This is a retrospective cohort study conducted at a large urban university teaching hospital ER in the United States located in the state of Missouri. The emergency room provides 24-hour emergency psychiatric consultation services by psychiatric residents, nurses, and attending physicians. In 2009 and 2010, this urban hospital emergency room received 54,047 patient visits, out of which 3886 were older adults (65+) and of those, 95 patients were geriatric patients who presented to the ER with a primary psychiatric complaint.

Data spanning two years, from January 2009 to December 2010, was gathered from review of electronic charts of patients meeting the three criteria: (1) age older than 65 years, (2) having a psychiatric disorder identified under the ICD code, and (3) presenting with a psychiatric complaint as their primary reason for visit. Subjects not meeting all three criteria were excluded. Subjects who did not present with a psychiatric complaint but were later diagnosed with a psychiatric condition were also excluded. Of the 95 patient charts meeting all 3 criteria, 20 patients had more than 1 visit, giving a total of 138 psychogeriatric patient visits.

### 2.2. Data Collection

All data were collected through chart reviews by the research team. A data collection sheet was employed to document selected demographic, psychosocial, and clinical features of the patients in the form of continuous and categorical variables. The variables included (1) gender, (2) age, (3) race, (4) location from which patient arrived and by whom the patient was accompanied, (5) reason for ER visit, (6) primary diagnosis obtained by ICD code, (7) specialists consulted, (8) discharge diagnosis from the ER, and (9) patient disposition. The reason for ER visit was established primarily using the ICD codes for that particular encounter for a subject and was secondarily confirmed and/or modified by reviewing the physician documentation of chief complaints. For nonspecific complaints like psychiatric problems, the reason for visit was established based on the history of present illness documentation. The major categories for the reason for emergency room visit were (1) anxiety, (2) delirium, (3) dementia, (4) mood disorder, (5) psychosis, and (6) others, with each category containing a subset of complaints.

Subjects were categorized based on the location from which they arrived as accompanied by family, arriving alone by emergency medical service, walk in, arriving from home, nursing home, arriving from jail, brought in by police, and transferred from outside hospital. The disposition data was categorized into discharge of subjects to home, nursing home, psychiatric unit, medicine unit, and others. The other category included critical care, surgery, neurology, nephrology, gastrointestinal unit, and cardiovascular unit.

### 2.3. Statistical Analysis

Data from the study were analyzed using SPSS 17.0. Data analysis included descriptive statistics with a report of the appropriate frequencies, means, and standard errors to describe the demographic and background characteristics of the study participants. Chi square tests were conducted to determine differences among multiple categorical independent and parametric dependent variables.

## 3. Results

Of the total subjects (*n* = 95) included in the study, about one third (33.7%) were males and two thirds (66.3%) were females with an average age of 75.5 years (S.E = ±0.81). Of the total visits (*n* = 138) females incurred majority (68.1%) of the visits. A breakdown by race indicated that Caucasians comprised a plurality (44.2%) of the patient population and were responsible for 40.1% of the visits. The vast majority of patients were African Americans (54.7%) who were responsible for 58.7% of the visits (Table 1).

As it relates to the mode of arrival, almost a third of the psychogeriatric patient visits were from a nursing facility (34.1%) and arrival from home unaccompanied by family via EMS (32.6%). Other major groups were walk-in patients unaccompanied by family (18.1%) and patients accompanied by family (11.6%) (Table 2). In addition, the reason for ER visit was categorized and majority (61.6%) of patients visited due to delirium. More than 1 in 10 visits were for anxiety disorder (16.7%) and mood disorders (12.3%) (Table 2).

Provision of psychiatric consultation was assessed based on race, gender, and diagnosis.  Table 3(a) illustrates the race and gender based differences in provision of psychiatric consultation in the ER. Caucasians (32.1%) were significantly more likely than African-American patients (8.6%) to get a psychiatric consult. Females (21.5%) were more likely than males (11.4%) to get a psychiatric consultation; however, the difference was not statistically significant. We compared the likelihood of receiving psychiatry consultations based on chief complaint. The vast majority of patients with delirium did not get a psychiatry consultation as compared to patients with other psychiatric complaints (97.6% versus 50.9%, *P* < 0.001) (Table 3(b)).

We compared the disposition for patient visits due to delirium with all patient visits. The disposition comprising the largest percentage of both categories was to the medicine inpatient unit—48.2% of delirium visits and 35.5% of all visits ended up in the medicine unit (Table 4). Only two (2.4%) delirium visits led to a disposition to the psychiatric inpatient unit while almost 1 in 6 (16.7%) of all other visits led to the psychiatric inpatient unit disposition. Patients who had a psychiatric inpatient unit disposition were significantly more likely to have had a psychiatric consultation in the ER as compared to patients who had other dispositions (*P* < 0.001). Patients who had a medicine inpatient unit disposition were significantly less likely to have a psychiatric consult in the ER as compared to patients who had other dispositions (*P* = 0.03).

We further explored the clinical data for the two delirium visits made by patients who were admitted to the psychiatric inpatient unit. The first visit was made by a patient who was on four psychotropic medications—an antidepressant, anxiolytic, antidementia drug, and a sedative-hypnotic. The second visit was by a patient who had previous psychiatric diagnoses of depression, schizophrenia, bipolar disorder, and obsessive compulsive disorder. Neither patient had electrolyte imbalance, urinary tract infection, or fever. In contrast, the median number of psychotropic medications of patients with delirium who were not admitted to psychiatry unit was “2” and the median number of previous psychiatric diagnoses was “2.” Of the delirium visits by patients that ended up in the medicine unit, 41% were on five or more classes of nonpsychotropic medications, 31% had elevated blood urea nitrogen (BUN) and creatinine, 28% had electrolyte imbalances, 6% had urinary tract infections, and 5% were febrile.

## 4. Discussion

The purpose of this study was to investigate the management and disposition of psychogeriatric patients in the ER as a function of variables such as gender, race, and chief complaint. Our study reports unique findings as opposed to some of the previous research investigations. An alteration in mental status was previously identified as a high risk for admission to an inpatient psychiatry unit and institutionalization after discharge [8]. Consistent with that finding, research by Wetterling and colleagues showed that delirium was identified as the primary reason for inpatient psychiatric admission [11]. In our study, we found that although delirium constitutes the main reason for ER visit (61.6%) in the older population of psychogeriatric patients, it was not the main reason for obtaining a psychiatric consultation in the ER and it was not the main reason for referral to a geriatric psychiatry inpatient unit. We found that patients with delirium, compared to those with other psychiatric illnesses, were actually less likely to get a psychiatry consult in the ER and were less likely to be admitted to an inpatient psychiatric unit. Subjects with reason for ER visit other than delirium, such as anxiety disorder, mood disorder, and psychosis, were more likely to get a psychiatric consult and were more likely to have a psychiatric disposition. Furthermore, patients with delirium as the presenting complaint were more likely to be transferred to inpatient medicine units and less likely to be transferred to a geriatric psychiatry inpatient unit compared to patients with any other diagnoses.

There are certain proposed explanations for our findings [11–14]. Patients with delirium may have been preferably transferred to the medicine inpatient unit because those patients were medically unstable for transfer to the geriatric psychiatry unit. Of the delirium visits by patients that ended up in the medicine unit, the largest proportion was on five or more classes of non-psychotropic medications. Polypharmacy in the elderly leads to higher rates of adverse drug effects due to age-related changes in pharmacodynamics. In addition to polypharmacy, patients transferred to the medicine unit also had elevated BUN and creatinine, electrolyte imbalances, urinary tract infections and were febrile. The fact that patients with these concomitant presentations were transferred to the medicine unit indicates that physicians prefer to treat these nonpsychiatric conditions there rather than in the psychiatric inpatient unit. The two patients with delirium who were admitted to the psychiatric unit did not have any concomitant presentations but were on more psychotropic medications and had more previous psychiatric diagnoses than the patients admitted to the medicine floor, indicating that physicians prefer to keep these patients in the psychiatric unit.

We propose that a well-equipped geriatric psychiatry unit with a well-trained physician and nursing staff can manage common illnesses such as urinary tract infections, dehydration, and electrolyte imbalance. The Geriatric Emergency Medicine Task Force recommends a mental status assessment for all elderly patients presenting to the ER [13]. Elderly patients coming to the ER with delirium caused by urinary tract infection, dehydration, or electrolyte imbalance may be routinely admitted to a well-equipped geriatric psychiatry ward. Such proposal is prudent given that delirium is a diagnosis for 14–56% of US elderly patients and is associated with hospital mortality rates of 22–76%. Also, in the case of delirium diagnosis, it is unrecognized in vast majority (66–70%) of the cases. Our proposal of a well-equipped geriatric psychiatric unit to manage cases of delirium may also be cost effective given the economic burden imposed by cases of delirium. Delirium in hospitalized older patients accounts for more than 49 percent of all hospital days, complicates hospital stays for at least one in five elderly patients who are hospitalized each year, and is responsible for billions of dollars spent as Medicare hospital expenditures [15].

One finding of critical concern was the race based difference in getting a psychiatric consultation. Caucasian patients were more likely to get a psychiatric consultation than minority patients in the ER. To determine whether this difference is due to more Caucasians having non-delirium visits, we found that even though Caucasian patients comprised 40.1% of all visits to the ER, they comprised only 25.9% of delirium visits and the rest were visits made by minority patients. This data shows that even though the difference in whether or not a patient obtains a psychiatric consult may not be due to race, it is more likely that Caucasians are presenting with more nondelirium visits which are better handled with a psychiatric consult. The reason that minority groups are more likely than Caucasians to present to the ER with delirium may be because of decreased access to healthcare on a regular basis, making the acute illness more prevalent. Another hypothesis is that minority patients may have multiple comorbidities which predispose them to delirium such as dementia, chronic kidney disease, end-stage liver disease, or terminal illness. Several other factors have been reported to be predictors of delirium in individuals and some of these may disproportionately affect minority elders. A few notable examples include visual impairment, baseline psychoactive drug use such as narcotics, benzodiazepines, and anticholinergics, history of alcohol abuse, and malnutrition.

We also found that females (21.5%) are more likely to get psychiatric consults than males (11.4%) even though the difference was not statistically significant. In order to validate this difference, we may need a larger sample of patients. If this difference were indeed proved to be real, this finding would be contrary to ER data in patients with acute myocardial infarction showing that men in the ER are more likely to get fibrinolytic treatment than women [14]. Overall, the most disconcerting finding was how only a few geriatric patients presenting to the ER with a psychiatric complaint as their primary complaint actually receive a psychiatry consultation.

There are limitations to our study. First, we included only those people who came to the ER with psychiatric complaints that fit into the 6 categories mentioned in our study method. It is likely that some of the older adults come in with nonspecific complaints and are found to have a psychiatric diagnosis as an end result of the ER investigations. We run the risk of missing that sample population due to our inclusion criteria. Second, our data set samples a select group of patients coming to the ER of a university teaching hospital which is located in a predominantly African-American community. African Americans comprise over 50% of the city's population and this demographic is not representative of the US population. Third, our study is a retrospective cohort study and cannot help establish cause and effect relationships.

## 5. Conclusion

The present study adds knowledge to the literature on diagnostic evaluation, disposition, and management of psychogeriatric patients in the ER. In elderly patients presenting with a primary psychiatric diagnosis, delirium is the chief complaint in a majority of visits to the ER and its cause is multifactorial. Although delirium has been a primary psychiatric diagnosis in the DSM since its formulation, it is a relevant finding in our study that only 2.4% of elderly patients that came to the ER with delirium as the presenting complaint had a psychiatric inpatient disposition, with the majority going to the medicine inpatient unit. Patients who went to the psychiatric ward were taking more psychotropic medications and had more previous psychiatric diagnoses than patients admitted to medicine. Compared with all psychogeriatric patients presenting to the ER, patients with delirium as the reason for visit also received significantly fewer psychiatric consultations. These findings show that the psychiatric inpatient unit is not believed to be as well-equipped to handle delirium patients as the medicine inpatient unit. We propose that a well-trained physician and nursing staff can manage delirium caused by urinary tract infections, dehydration, and electrolyte imbalance and that ER physician may routinely admit these patients to the geriatric psychiatry ward. Future studies can focus on ER physicians' perception and knowledge about psychiatric illnesses in the elderly, criteria for obtaining psychiatric consults, use of screening tools for psychiatric illnesses, and objective determinants of disposition of psychogeriatric patients.

## Figures and Tables

**Table 1:** Selected demographic characteristics of the patients.

	Total subjects	Total visits
	(*N* = 95)	(*N* = 138)
	*N* (%)	*N* (%)
Gender		
Males	32 (33.7)	44 (31.9)
Females	63 (66.3)	94 (68.1)

Race		
Caucasian	42 (44.2)	56 (40.1)
African American	52 (54.7)	81 (58.7)
Hispanic	1 (1.1%)	1 (0.7)

Age	Mean (±S.E): 75.5 years (±0.81)	

**Table 2:** Patient source and reason for emergency room visit.

Location/accompanied by	*N* (%)
Arriving from nursing facility	47 (34.1)
Arriving from home unaccompanied by family via emergency medical service (EMS)	45 (32.6)
Walk-in unaccompanied by family	25 (18.1)
Accompanied by family	16 (11.6)
Accompanied by police/arriving from jail	3 (2.1)
Arriving from another hospital	2 (1.4)

Reasons for emergency room visit (diagnosis)	*N* (%)

Delirium	85 (61.6)
Anxiety disorder	23 (16.7)
Mood disorder	17 (12.3)
Psychosis	6 (4.3)
Dementia	2 (1.4)
Other	5 (3.6)

Total visits (*N*) = 137.

**Table:** (a)

Psychiatry consultation	Race		*P* value	Gender		*P* value
	Caucasian (*N* = 56)	Afr. Amer. (*N* = 81)		Female (*N* = 93)	Male (*N* = 44)	
	*N* (%)	*N* (%)		*N* (%)	*N* (%)	
Obtained	18 (32.1)	7 (8.6)	0.03*	20 (21.5)	5 (11.4)	0.06
Not obtained	38 (67.9)	74 (91.2)		73 (78.5)	39 (88.6)	

Total visits (*N*) = 137. Afri. Amer. = Indicates African American patients. *P value = considered statistically significant if <0.05.

**Table:** (b)

Psychiatry consultation	Delirium (*N* = 85)	Other psychiatric complaints (*N* = 53)	*P* value
	*N* (%)	*N* (%)	
Obtained	2 (2.4)	26 (49.1)	<0.001*
Not obtained	83 (97.6)	27 (50.9)	

Total visits (*N*) = 138. *P value = considered statistically significant if <0.05.

**Table 4:** Disposition of patients who presented with delirium compared with all patients.

Disposition	Delirium visits only	All visits
	(*N* = 85)	(*N* = 138)
	*N* (%)	*N* (%)
Medicine inpatient unit*	41 (48.2)	49 (35.5)
Home	14 (16.5)	25 (18.1)
Nursing home	10 (11.8)	13 (9.4)
Psychiatric inpatient unit*	2 (2.4)	23 (16.7)
Other	18 (21.2)	28 (20.3)

*In this case, difference between groups was significant. *P* value = considered statistically significant if <0.05.
